# Notch1 Signaling Contributes to Hypoxia-induced High Expression of Integrin β1 in Keratinocyte Migration

**DOI:** 10.1038/srep43926

**Published:** 2017-03-07

**Authors:** Di Tang, Tiantian Yan, Junhui Zhang, Xupin Jiang, Dongxia Zhang, Yuesheng Huang

**Affiliations:** 1 Institute of Burn Research, State Key Laboratory of Trauma, Burns and Combined Injury, Southwest Hospital, The Third Military Medical University, Chongqing, China

## Abstract

Oxygen tension is an important micro-environmental factor that affects epidermal development and function. After injury, high oxygen consumption and vascular injury result in partial hypoxia. However, whether hypoxia benefits or hurts wound healing remains controversial. In this study, a tissue oxygen tension monitor was used to detect the spatial and temporal distribution of oxygen in burn wounds. *In vitro*, we demonstrate that hypoxia promoted the expression of integrin β1 and the migration of keratinocytes. Furthermore, hypoxia-induced migration was slowed by Notch1 ligands and a siRNA against ITGB1 (integrin β1). Our findings suggest that integrin β1 may be an oxygen-sensitive molecule that promotes keratinocyte migration during wound healing and that Notch1 signaling is involved in this process.

Wound healing is a very complex process that consists of many cellular and non-cellular components^1^. Oxygen tension, a key component of the epidermal micro-environment, is responsible for regulating many processes, including metabolic reactions, enzymatic reactions, and signal transduction^2^. Low oxygen tension, referred to as hypoxia, was previously thought to be only detrimental. However, it has recently been shown to play some useful roles in wound healing^3^,^4^,^5^. Although almost all wounds are accompanied by changes in oxygen tension^6^,^7^,^8^, the timing and extent of hypoxia in different wounds have not yet been fully explored^4^,^5^. Many previous studies have used hypoxia-inducible factor (HIF) as an indicator of hypoxia^9^,^10^,^11^, while others have used hypoxyprobe^12^ or pimonidazole-1 staining^13^. Regarding the duration of hypoxia, opinions differ, and the extent of hypoxia is often difficult to quantify. Thus, to determine the mechanisms underlying dynamic changes in oxygen tension after an injury, a more precise method for measuring oxygen tension in the wound region is needed.

Keratinocyte migration is a critical process during wound healing. Hypoxia accelerates the migration of keratinocytes^14^,^15^, but the mechanism underlying this acceleration is unclear. Integrin β1 is a member of the integrin family that mainly regulates cell-to-cell and cell-to-ECM (extra-cellular matrix) adhesion^16^. Previous study showed that integrin β1 had a crucial role in keratinocyte migration and wound re-epithelialisation^17^. Conditional ablation of integrin β1 in skin severe defects in epidermal proliferation, basement membrane formation, and hair follicle invagination^18^. However, the function of integrin β1 in hypoxia-induced keratinocytes migration remains unclear.

Notch1 signaling is a highly conserved pathway that is widely involved in cell fate decisions. Depending on the cell type and context, Notch1 signaling can either induce differentiation or maintain cells in an undifferentiated state^19^,^20^. Binding by ligands in the Delta and Jagged families induces the proteolytic cleavage of Notch receptor, resulting in an extracellular domain, a transmembrane domain and a Notch intracellular domain (NICD) that is then able to translocate to the nucleus. NICD translocation is used as an indicator of activated Notch signaling^19^. Previous studies have reported that the activation of Notch1 signaling induces epidermal differentiation and reduces the expression of integrins^21^,^22^,^23^. Moreover, Notch1 signaling has been shown to interact with hypoxia signaling pathways^24^,^25^,^26^. These data suggest that Notch1 signaling may play a role in hypoxia-induced migration.

In this study, we measured oxygen tension changes during wound healing and how these changes affect integrin β1 expression and keratinocyte migration. We observed that oxygen tension was significantly different between normal skin and wound edge. This difference coincided with a decrease in Notch1 signaling and an increase in integrin β1 comparing the wound edge with normal skin. We demonstrate that under hypoxic conditions, the high expression of integrin β1 and the accelerated migration of keratinocytes was reversed by Notch1 ligands (Jagged-1 or Dll4). Thus, integrin β1 may be an oxygen-sensitive molecule that promotes keratinocyte migration during wound healing and that Notch1 signaling is involved in this process.

## Results

### Spatiotemporal distribution of the oxygen tension during wound healing

**Figure 1:**
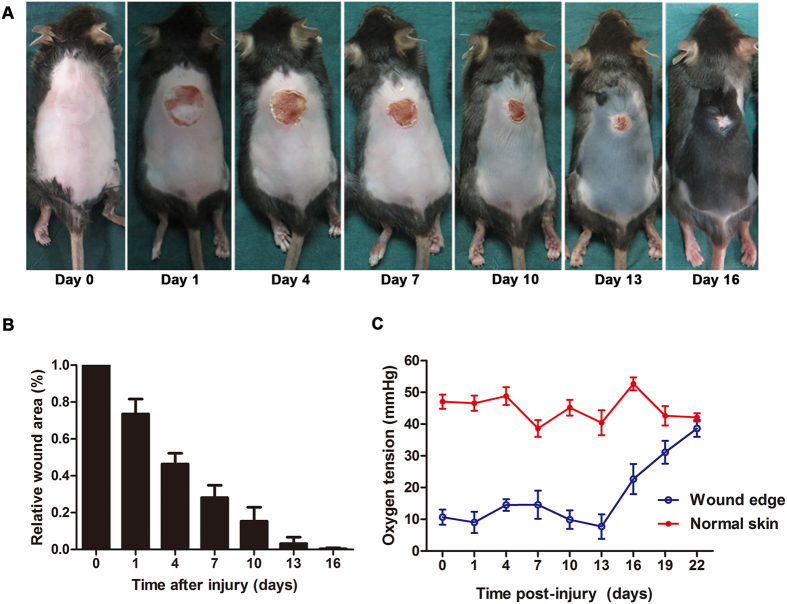
Spatiotemporal distribution of the oxygen tension during wound healing. An electrical scald instrument was applied at a constant temperature (80 °C) and pressure (0.5 kg) for 5 seconds to establish deep partial-thickness scald wounds with an area of 2.0 cm^2^. (**A**) Images of a representative mouse taken immediately post-injury (day 0) and on days 1, 4, 7, 10, 13, and 16. (**B**) The relative wound area is shown at the indicated time points. Values represent the means ± S.D. (n = 6 mice). (**C**) The spatiotemporal distribution of the oxygen tension during scald wound healing (n = 5 mice per time point).

A tissue oxygen tension monitor was used to detect the spatial and temporal distribution of oxygen in scald wounds. Oxygen tension at the wound edge was lower than in normal skin beginning the day immediately post-injury. The wound completely closed on the 16^th^ day (Fig. 1A,B). Interestingly, oxygen tension at the wound edge began rising at approximately the same time (Fig. 1C) and eventually returned to normal by the 22^nd^ day.

### Involvement of integrin β1 and Notch1 signaling in wound healing

**Figure 2:**
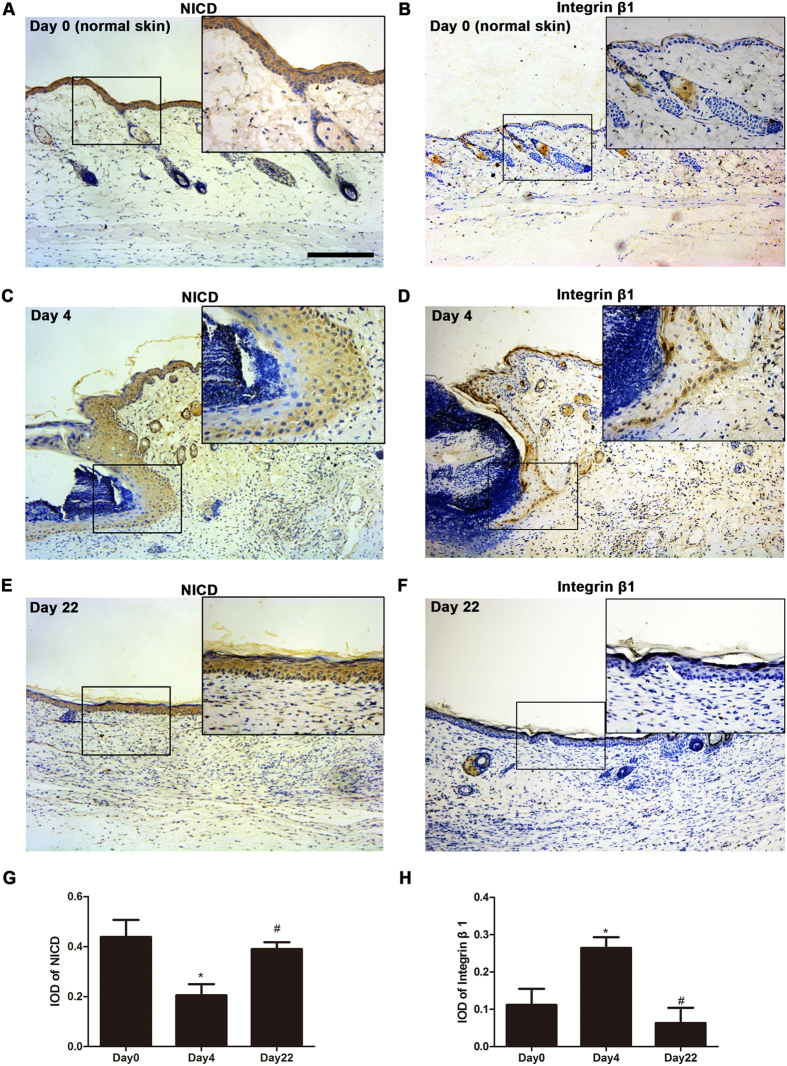
IHC showing the expression of integrin β1 and NICD on day0 (normal skin), day 4 (post-injury) and day 22 (the time point when oxygen tension back to normal) at the wound site. Images of skin tissue sections that were immunohistochemically stained for NICD. The tissues showed significantly lower levels of NICD (**C**) at the wound edge than those were observed (**A**) in normal skin. Images of skin tissue sections stained for integrin β1 showing that higher levels of integrin β1 were expressed (**D**) at the wound edge than (**B**) in normal skin. Both NICD and integrin β1 levels came back to normal (**E**,**F**) on day22. Scale bar indicates 200 μm. (**G**,**H**) The corresponding Integral optical density (IOD) for NICD and integrin β1 was measured at the indicated times. Values represent the means ± S.D. (n = 3~6 sections per group). *P < 0.05 versus normal skin (day 0 group); ^#^P < 0.05 versus wound edge (day 4 group).

Our findings show that NICD, an indicator of activated Notch1 signaling, was expressed at lower levels in wound edge (Figs 2C and S1) than in normal skin (Fig. 2A). Coincidentally, the expression of integrin β1 was significantly higher in the wound edge (Figs 2D and S1) than in normal skin (Fig. 2B). After the oxygen tension returned to normal on day 22, the expression of NICD was increased (Fig. 2E) and the expression of integrin β1 was decreased (Fig. 2F). The change tendencies (Fig. 2G,H) indicate that changes in oxygen tension may be the reason for the difference in the expression of integrin β1 and NICD during wound healing.

### Low oxygen tension (2% O_2_) increased the expression of integrin β1 concomitantly with the inactivation of Notch1 signaling *in vitro*

**Figure 3:**
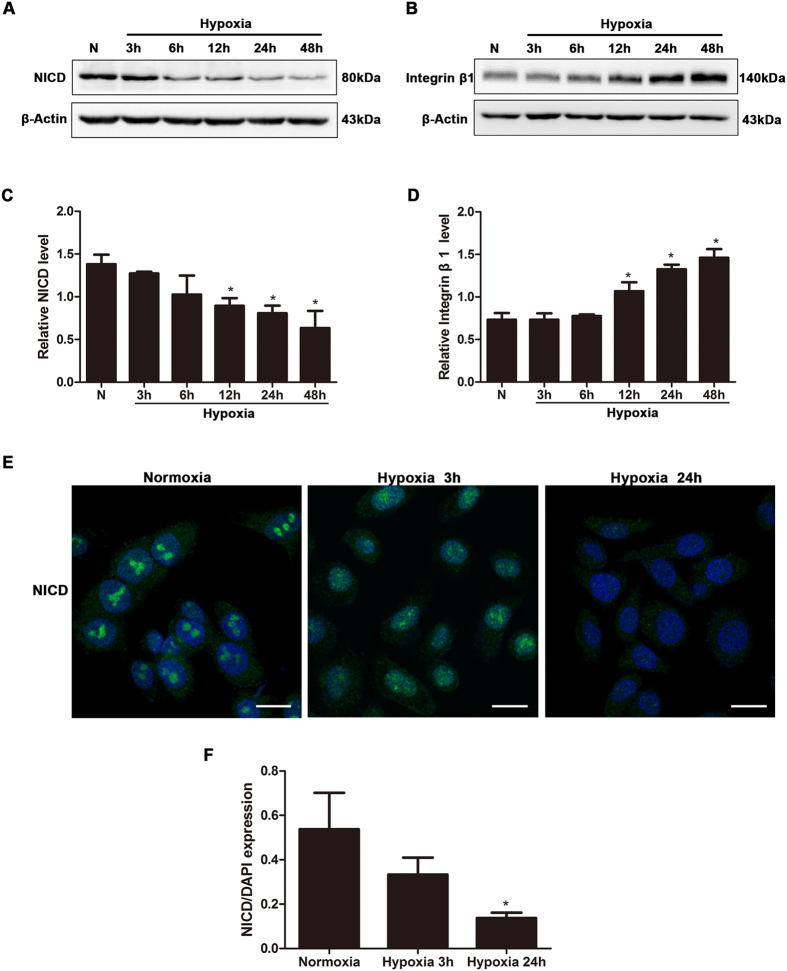
The effect of hypoxia on NICD and integrin β1 expression. HaCaT cells were incubated in a normal or hypoxia chamber for 3 h, 6 h, 12 h, 24 h, or 48 h. (**A**,**B**) Western blot analysis showed that as exposure to hypoxia continued, NICD expression decreased, while integrin β1 expression increased. The image shown is representative of three independent experiments. (**C**,**D**) Quantitative analysis of NICD and integrin β1 expression levels under normoxic conditions or after exposure to hypoxia for the indicated times. Values represent the means ± S.D. of three independent experiments. N, normoxia; *P < 0.05 versus the N group. (**E**) Immunofluorescence staining for NICD (green) in HaCaT cells exposed to normoxia or treated with hypoxia for the indicated times. Nuclei were stained using DAPI (blue). Scale bar = 20 μm. (**F**) A graph is shown to illustrate changes in NICD expression levels under normoxic conditions and in cells exposed to hypoxia for the indicated times. The corresponding Integral optical density (IOD) for NICD (green) values was normalized to that of DAPI (blue). Values represent the means ± S.D. of three independent experiments. *P < 0.05 versus the Normoxia group.

To study the effect of hypoxia on the expression of NICD and integrin β1, we established a cellular hypoxia model. The oxygen tension in the wound edge was approximately 15 mmHg (Fig. 1C), while atmospheric pressure was 760 mmHg. We therefore selected 2% O_2_ to induce hypoxia, and we detected HIF-1α as positive control showing that this hypoxia condition effectively works (Fig. S2). In HaCaT cells, Western blot analysis showed that exposure to hypoxia decreased the expression of NICD (Fig. 3A,C) and Notch1 signaling downstream molecular Hes1 (Fig. S3A), but strongly increased the expression of integrin β1 (Fig. 3B,D). These *in vitro* results are in agreement with the previously observed *in vivo* phenomenon. Moreover, immunofluorescence staining for NICD (green) showed that hypoxia suppressed both the expression of NICD (Fig. 3F) and the translocation of NICD from the cytoplasm to the nucleus (Fig. 3E), which is another indicator of activated Notch1 signaling. These data demonstrate that low oxygen tension caused the observed increase in the expression of integrin β1 and the suppression of Notch1 signaling.

### Effect of Notch1 signaling on the hypoxia-induced increase in the expression of integrin β1

**Figure 4:**
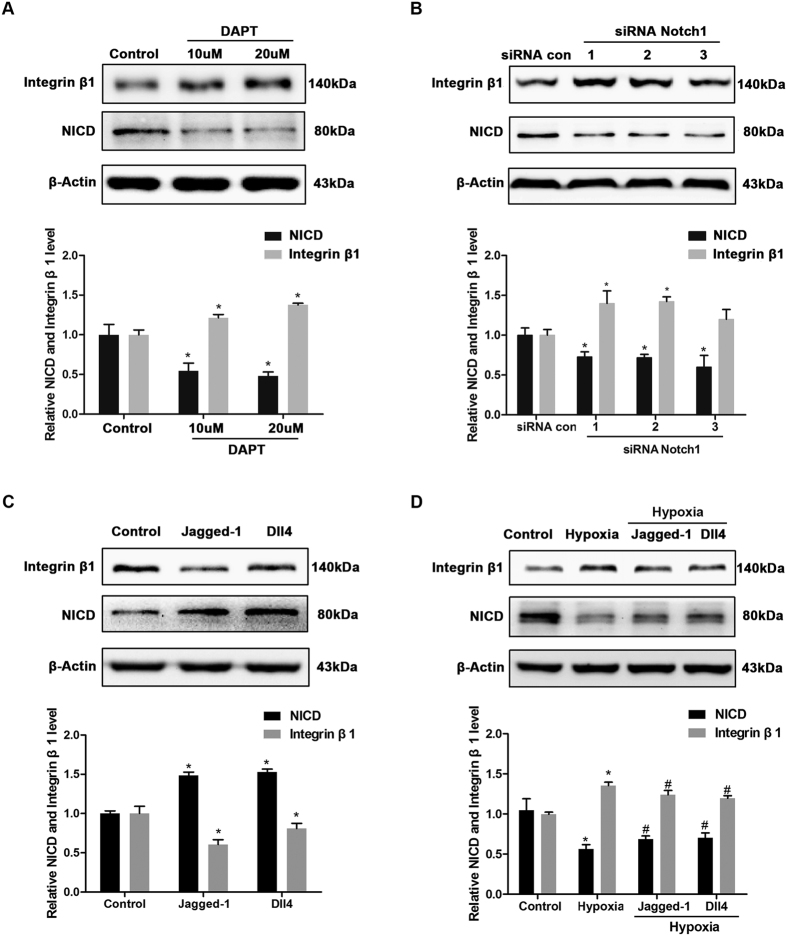
The effect of regulating Notch1 signaling on the expression of integrin β1. (**A**) Western blot and a quantitative analysis showed that exposure to DAPT (10 μM or 20 μM, 24 h) decreased the expression of NICD and increased the expression of integrin β1. (**B**) Western blot and a quantitative analysis showed that the expression of NICD was down-regulated and the expression of integrin β1 was up-regulated after interference of Notch1 compared with a negative control (siRNA con). SiRNA interference lasted 6 h, and then cells were cultured for 24 h before harvest. (**C**) Western blot and a quantitative analysis showed that Jagged-1 (1 μg/ml, 24 h) or Dll4 (1 μg/ml, 24 h) effectively activated Notch1 signaling and reduced the expression of integrin β1 in normoxia. (**D**) Western blot and a quantitative analysis showed that Jagged-1 (1 μg/ml, 24 h) or Dll4 (1 μg/ml, 24 h) effectively reduced the hypoxia-induced increase in the expression of integrin β1. Values represent the means ± S.D. of three independent experiments. Values were normalized after comparison with control group or siRNA con group. *P < 0.05 versus corresponding column in the control group or siRNA con group; and ^#^P < 0.05 versus corresponding column in the Hypoxia group.

To determine whether Notch1 signaling mediates the hypoxia-induced increase in the expression of integrin β1, we used the Notch1 ligands (Jagged-1 or Dll4), Notch1 inhibitor DAPT and siRNA Notch1 to regulate Notch1 signaling. Western blot analysis showed that DAPT increased the expression of integrin β1 in a dose-depending manner (Fig. 4A). SiRNA Notch1 effectively down-regulated NICD (Fig. 4B) and Hes1 (Fig. S3B), and increased integrin β1 through specific interference with Notch1.

Under normoxia condition, Jagged-1 and Dll4 both effectively activated Notch1 signaling, and decreased the expression of integrin β1 (Fig. 4C). More importantly, under hypoxia condition Jagged-1 and Dll4 both restored Notch1 signaling (Fig. S3C) and suppressed the hypoxia-induced increase in the expression of integrin β1 (Fig. 4D). These data suggest that Notch1 signaling may play an important role in mediating the hypoxia-induced increase in the expression of integrin β1. Given that integrin β1 plays an important role in the regulation of cell migration and our previous finding that the low oxygen tension lasts throughout the re-epithelializing process (Fig. 1C), we hypothesized that Notch1signaling and integrin β1 expression may contribute to hypoxia-induced keratinocyte migration.

### Effect of Notch1 signaling and integrin β1 on hypoxia-induced keratinocyte migration

**Figure 5:**
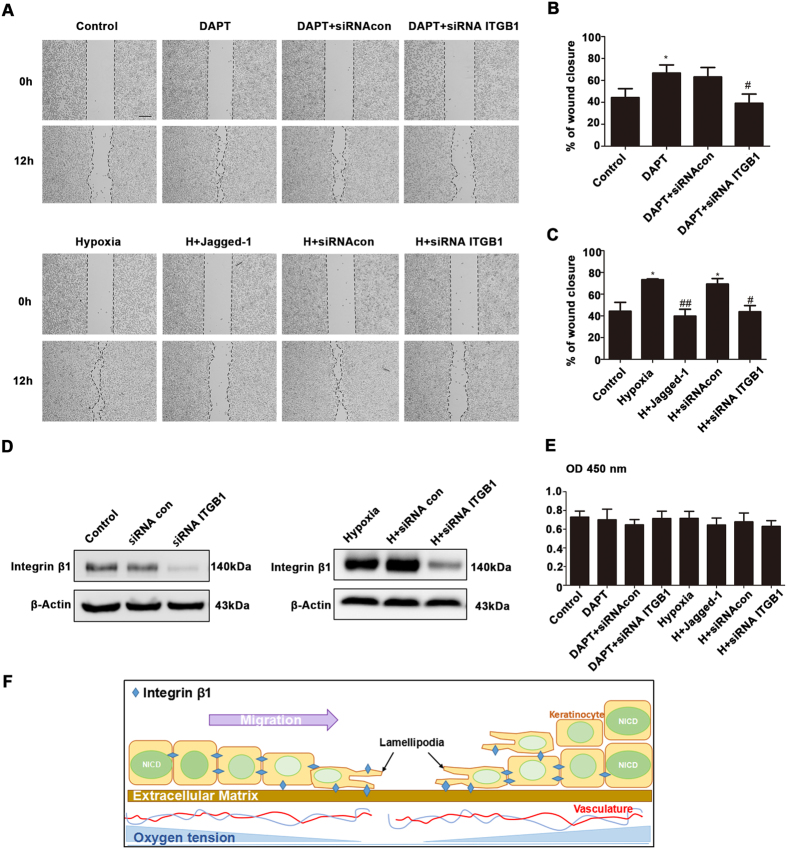
The hypoxia-induced up-regulation of integrin β1 promoted HaCaT cells migration. (**A**) The following experiments are shown: HaCaT cells exposed to no treatment (Control), integrin β1 was upregulated following the inhibition of Notch1 signaling using DAPT (DAPT), integrin β1 was upregulated by low oxygen tension (Hypoxia), cells transfected with a negative control (siRNAcon) or a siRNA against integrin β1 (siRNA ITGB1) were scratch-wounded using Culture-Inserts, whose width of cell-free gap is 500 μm. The results were recorded using a phase-contrast microscope connected to a digital camera from time 0 to 12 h (n = 4 independent experiments). Bar = 200 μm. Before 12 h monitoring, each group was treated with indicated treatment for 12 h. As to siRNA groups, 6 h for interference, 12 h cultured under indicated condition. (**B**,**C**) Wound closure was demonstrated by determining the area covered by keratinocytes immediately after wounding and 12 h later. Each panel represents the wound closure level of each group. The results were calculated by measuring the reduction in the wound bed surface over time using Image J software. *P < 0.05 versus control group. ^#^P < 0.05 versus the group including siRNA con. ^##^P < 0.05 versus hypoxia group. (**D**) Western blot showing the expression of integrin β1 after interference with siRNA ITGB1 in HaCaT cells under normoxia (left panel) and hypoxia (right panel) conditions. (**E**) HaCaT cell proliferation was analyzed using Cell Counting Kit-8 (n = 4 independent experiments). (**F**) Schematic model of oxygen tension tightly regulating the expression of integrin β1 and keratinocyte migration through Notch1 signaling. After injury, high oxygen consumption and vascular injury result in partial hypoxia. Low oxygen tension in wound edge induces keratinocytes to express integrin β1 via inhibition of Notch1 signaling. Integrin β1, as a lamellipodia protein, improves keratinocytes migration during the re-epithelization process.

Both inactivation of Notch1 signaling (DAPT or siRNA Notch1) and hypoxia treatment significantly promoted the migration of HaCaT cells in a scratch wound model (Figs 5A–C and S4A–C). When a siRNA against integrin β1 (siRNA ITGB1) was added to cells, cell migration was significantly impaired (Figs 5B,C and S4D). Moreover, cell migration assays also showed that the Notch1 ligands (Jagged-1 or Dll4) significantly suppressed the hypoxia-induced increase in the migration of HaCaT cells (Figs 5C and S4B). Transfecting siRNA ITGB1 was effective (Figs 5D and S4E,F). There was no detectable difference in cell proliferation among these groups (Figs 5E and S4G–I). Thus, cell proliferation can be excluded as a possible explanation for alterations in migratory capacity. Our findings therefore suggest that Notch1 signaling and integrin β1 participate in hypoxia-induced keratinocyte migration. This leads us to propose a model of oxygen tension tightly regulating the expression of integrin β1 and keratinocyte migration through Notch1 signaling (Fig. 5F).

## Discussion

In the current study, we first used the OxyLite^TM^ system to demonstrate the spatiotemporal distribution of the oxygen tension during the wound healing process. We found that oxygen tension was significantly different between normal skin and wound edge. We also demonstrated that low oxygen tension promoted integrin β1 expression and keratinocyte migration, and these effects were reversed by Notch1 ligands (Jagged-1 or Dll4) and a siRNA against integrin β1 *in vitro*. Our findings suggest that integrin β1 is a main effector molecule sensing the change in oxygen tension, and that integrin β1 promotes wound healing by accelerating the migration of keratinocytes. Additionally, we show that Notch1 signaling is involved in this process.

Hypoxia is a very important microenvironmental factor that accompanies wound healing^11^,^27^. Because there is currently no way to precisely detect hypoxia in a wound, it has historically been difficult to quantify the extent of hypoxia during wound healing. The OxyLite^TM^ system is based on the principle of fluorescence quenching. This device provides many advantages over former methods, which have included real-time monitoring, detecting the large potential range of dissolved oxygen (0–200 mmHg or 0–25% O_2_ concentration), the constant monitoring of tissues unable to consume oxygen, and outputs that came in the form of absolute values of oxygen tension. Our findings show that the oxygen tension in wound edge is approximately 10 mmHg (equivalent to 2% O_2_) and that hypoxia emerges immediately after an injury and is constantly present until the wound closes (Fig. 1C). Some of the differences in opinion regarding the time points at which oxygen tension changes^13^ may be because of that Pimonidazole is sensitive only at a pO_2_ of less than 10 mmHg and that it requires time to take effect. Given that low oxygen tension accompanies by the re-epithelization process (Fig. 1), hypoxia may play a critical role in the keratinocyte migration.

In addition to lower oxygen tension, our *in vivo* experiments also showed that Notch1 signaling was silenced and integrin β1 expression was increased at the wound edge (Figs 2 and S1). Integrin β1 is normally expressed in the basal layer and in hair follicle bulges, but after an injury, it is highly expressed and abnormally distributed (Fig. S1). This phenomenon may due to the fact that alpha and beta-1 integrins may play unique roles in migration^17^,^18^.

Notch1 signaling is down-regulated in disorganized proliferating epidermis, such as that observed in carcinoma and psoriasis and during the first step of re-epithelialization, but it then returns to normal levels in psoriatic plaques following treatment with phototherapy as well as in newly regenerated stratified epidermis following wound healing^28^. Our observations are in agreement with this phenomenon (Fig. 2G). Interestingly, we found that variation in Notch1 signaling occurred in parallel with the changes we observed in oxygen tension after injury (Fig. 1C). We therefore suggest that Notch1 signaling is associated with an oxygen-sensing pathway.

In our *in vitro* study, we chose a 2% oxygen concentration to simulate a hypoxic environment because it is similar to the environment caused by oxygen tension in the wound edge. HaCaT, a human keratinocyte cell line, is a traditional cell model that is routinely used to study re-epithelization in wound healing. We convincingly demonstrated that in HaCaT cells, as exposure to hypoxia continued, the expression of NICD was significantly down-regulated, while integrin β1 expression and keratinocyte migration were both up-regulated. Moreover, exogenously inhibiting Notch1 signaling (DAPT or siRNA Notch1) resulted in the same effect as was provoked by hypoxia (Figs 4 and 5).

Canonical Notch1 signaling is a switch that occurs in the epidermal lineage that commits a cell to differentiating and decreases the expression of integrins^1^,^22^. These results provide evidence supporting our observations. However, other studies have shown that in CHO (Chinese hamster ovary) cells NICD activates integrins^29^ and that in vascular endothelial cells and fibroblasts NICD promotes migration^30^. These studies indicate that Notch1 activation produces diverse cellular effects in a cell-type and context-dependent manner^1^,^29^,^31^,^32^. In keratinocytes, we found that hypoxia-induced inhibition of Notch1 signaling regulated integrin β1 expression, but the mechanism underlying the interaction between Notch1 signaling and integrin β1 remains unclear. Future studies are expected to reveal the details of this process.

Taken together, our results illustrate that Notch1 signaling contributes to the hypoxia-induced up-regulation of integrin β1 at the wound edge, and the high expression of integrin β1 promotes keratinocyte migration (Fig. 5F). While oxygen’s role in mediating wound healing has been recognized for decades^6^,^7^, the importance of cellular oxygen sensing in cellular adaptive and reparative pathways is a relatively new area of research^3^,^4^,^11^. The results of this study potentially introduce a new viewpoint that increases our understanding of the underlying mechanisms that surround the biological functions of hypoxia in keratinocytes.

## Methods

### Ethics Statement

All animal-based investigations were designed and performed in accordance with the Guide for the Care and Use of Laboratory Animals published by the National Institutes of Health (NIH Pub. No. 85–23, revised 1996). The entire project was reviewed and approved by the Animal Experiment Ethics Committee of the Third Military Medical University in Chongqing, China.

### Mouse wound-healing experiments and immunohistochemistry

An electrical scald instrument was applied for 5 seconds at a constant temperature (80 °C) and pressure (0.5 kg)^33^ to establish a scald wound with an area of 2.0 cm^2^ on the backs of C57BL/6 mice. On the indicated days, digital photographs were taken of the injury site in which a standard-sized dot was placed beside the wound. Wound size was expressed as the ratio of the wound area to the dot measurement. An OxyLite^TM^ system was applied to measure oxygen tension in the skin on days 0 and 1 after injury and then every three days until day 22, when the oxygen tension of wounds had returned to normal. Wounded areas surrounded by unwounded skin were dissected from the animals on days 0 (normal skin), 4, 10, and 16 after injury. The skin specimens were then fixed in paraformaldehyde, embedded in paraffin, and sectioned. Sectioned wound specimens were deparaffinized and rehydrated. Antigen retrieval was performed by heating the sections in citrate buffer (pH 6.0) in a microwave at 650 W for 6 minutes. To perform immunofluorescence staining for NICD and integrin β1, wound specimens were incubated with rabbit anti-activated Notch1 (1:100 dilution; Abcam, UK) or rabbit anti-integrin β1 (1:100 dilution; Abcam, UK) primary antibodies at 4 °C overnight. The specimens were then washed and incubated with biotinylated secondary antibodies (1:500 dilution; GE Healthcare Life Sciences, USA) and streptavidin-HRP (1:500 dilution; Zymed Laboratories, USA). Color was developed using DAB peroxidase substrate (DAKO, Denmark) until an optimal color was observed.

### Cell culture and reagents

HaCaT cells were obtained from the Cell Bank of the Chinese Academy of Sciences in Beijing, China. Cells were cultured in RPMI 1640 medium (HyClone, USA) supplemented with 100 U/ml penicillin, 100 mg/ml streptomycin, and 10% fetal bovine serum (HyClone, USA). The cells were incubated at 37 °C in 5% CO_2_, and 95% humidity. DAPT was obtained from Selleck Laboratories (Cat. No. S2215, USA). The recombinant human Jagged-1 protein (Cat. No. Ab 108575, UK) and Dll4 protein (Cat. No. Ab 108557, UK) were purchased from Abcam.

### Hypoxia treatment

Hypoxic conditions were created using a Forma Series II Water Jacket CO_2_ incubator (model: 3131; Thermo Scientific), with which we could precisely control oxygen levels and temperature. The hypoxic conditions were generated at 37 °C in 5% CO_2_ and the designated oxygen content was balanced with N_2_. All of the media used in the hypoxia experiments were preincubated overnight in the chambers with the designated oxygen content.

### Immunofluorescence staining

Cells were cultured on glass coverslips and fixed in 4% paraformaldehyde for 20 min. The fixed cells were then incubated with rabbit anti-activated Notch1 (1:100 dilution; Abcam, UK) primary antibodies overnight at 4 °C and then washed with PBS. The cells were then incubated with secondary antibodies conjugated to FITC (1:100 dilution; Beyotime, Shanghai, China) at 37 °C for 1 h. Nuclei were stained using DAPI (HyClone, USA). The expression of NICD was analyzed using a Leica Confocal Microscope (Leica Microsystems, Wetzlar, Germany).

### Western blot analysis

The cells were washed with ice-cold phosphate-buffered saline (PBS) and harvested on ice. The protein concentration of the resulting lysates was determined using a RCDC protein assay kit (Sigma, USA). A prestained standard protein molecular weight marker and the protein samples were loaded into wells and separated using 8% SDS–PAGE. The blots were then transferred using electroblotting to polyvinylidene difluoride (PVDF) membranes. After the membranes were blocked with 5% nonfat dried milk or bovine serum albumin (BSA), the desired bands were incubated overnight at 4 °C with the corresponding primary antibodies, including rabbit anti-activated Notch1 (1:1000 dilution; Abcam, UK), rabbit anti-integrin β1 (1:1000 dilution; Abcam, UK), rabbit anti-HIF-1α (1:1000 dilution; Proteintech, USA) and mouse anti-β actin (1:1000 dilution; Santa Cruz, USA). Horseradish peroxidase-conjugated secondary IgG (1:5000 dilution; Proteintech, USA) was subsequently used as the secondary antibody. The results were analyzed using a ChemiDoc imaging system (Bio-Rad, USA).

### SiRNA transfection

To specifically inhibite Notch1 signaling, siRNA Notch1 and a negative control siRNA containing non-specific siRNA (siRNAcon) were purchased from Shanghai GenePharma, Co. Ltd (Shanghai, China). To knock down integrin β1, siRNA ITGB1 and siRNAcon were also purchased from Shanghai GenePharma, Co. Ltd (Shanghai, China). HaCaT cells were transfected with siRNA Notch1, siRNA ITGB1 or siRNAcon for 6 h according to the manufacturer’s protocol, and then these cells were cultured in RPMI 1640 medium for 24 h. Transfection efficiency was detected using a fluorescence microscope (Leica Microsystems, Wetzlar, Germany).

### Cell scratch wound assays

The scratch wound assay consisted of an *in vitro* incisional wound model and was performed as previously described^34^. To standardize wound sizes, Culture-Inserts (Ibidi, Germany) were used to form the scratch wounds. A 70 μl volume of 7 × 10^4^/ml cell suspension was added to each well containing a Culture-Insert. An appropriate time was allowed for cell attachment (24 h). Then cells were received with respective treatment (12 h). Next the cells were incubated at 37 °C for 2 h in the presence of mitomycin-C (5 μg/ml) to inhibit cell proliferation. After the Culture-Insert was removed, a cell-free gap of 500 μm was created. During the next 12 h, cell migration was evaluated in each well. Transfection efficiency was evaluated under an inverted phase contrast microscope, and the results were analyzed using NIH Image J software.

### Cell proliferation assay

HaCaT cells were seeded at 2 × 10^3^ cells/well in 96-well plates. Cell proliferation was analyzed using Cell Counting Kit-8 (CCK-8; Beyotime, China) according to the manufacturer’s instruction. After inoculating the cell suspensions in 96-well plates, the plates were pre-incubated for 24 h in a humidified incubator at 37 °C in 5% CO_2_. Different treatments consisting of the indicated conditions were applied for 12 h. To maintain consistent conditions during the cell scratch wounding assay, mitomycin-C (5 μg/ml) was also applied for 2 h. After 12 h, the CCK-8 solution (10 μl) was added to each well of the plate, and the plate was then incubated for 1 h. Finally, absorbance was measured at 450 nm using a microplate reader (Thermo, USA).

### Statistical analysis

The data are presented as the mean ± standard deviation (SD). SPSS 13.0 was used for the statistical analysis, the statistical significance of differences among multiple groups was evaluated using one-way ANOVA. A P value < 0.05 was considered to indicate statistical significance.

## Additional Information

**How to cite this article:** Tang, D. *et al*. Notch1 Signaling Contributes to Hypoxia-induced High Expression of Integrin β1 in Keratinocyte Migration. *Sci. Rep.* **7**, 43926; doi: 10.1038/srep43926 (2017).

**Publisher's note:** Springer Nature remains neutral with regard to jurisdictional claims in published maps and institutional affiliations.

## Supplementary Material

Supplementary Information
