# Supplementary material for: Notch1 Signaling Contributes to Hypoxia-induced High Expression of Integrin β1 in Keratinocyte Migration
Source: Sci Rep. 2017 Mar 7;7:43926. doi: 10.1038/srep43926 (PMC5339698; doi:10.1038/srep43926)
Supplement: Supplementary Information [file srep43926-s1.pdf]

Notch1 Signaling Contributes to Hypoxia-induced High Expression of Integrin β1 in Keratinocyte Migration

Di Tang[1], Tiantian Yan[1], Junhui Zhang[1], Xupin Jiang[1], Dongxia Zhang[1], Yuesheng Huang[1,*]

[1]Institute of Burn Research, State Key Laboratory of Trauma, Burns and Combined Injury, Southwest Hospital, The Third Military Medical University, Chongqing, China.

[*]Correspondence and requests for materials should be addressed to Yuesheng Huang.

Email address: yshuangtmmu @163.com

Institute of Burn Research, State Key Laboratory of Trauma, Burns and Combined Injury, Southwest Hospital, The Third Military Medical University, Chongqing 400038, China.

Tel: +86-23-68766023

Supporting Information

Supplemental Figure Legends

Figure S1 IHC showing the expression of integrin β1 and NICD in wound edge on day4. In every row, the first picture is showing the structure and the following two pictures are higher magnification photographs showing the details of the first picture. Scale bar indicates 200 μm. In every sample, in order to show the relationship between integrin β1 and NICD, the corresponding parts were chosen to take photos.

Figure S2 Western blot results of HIF-1α showing the hypoxia condition effectively worked. (A) Western blot and a quantitative analysis showed that as exposure to hypoxia continued, expression of HIF-1α was constantly higher than N (normoxia) group, which indicating hypoxia treatment effectively worked. (B) Western blot and a quantitative analysis showed that with or without the regulation of Notch1 signaling, expression of HIF-1α was higher than control group, which indicating hypoxia treatment worked well.

Figure S3 The effect of hypoxia and regulating Notch1 signaling on the expression of Hes1. (A) Western blot showed that exposure to low oxygen tension (2% O_2, 24 h) decreased the expression Hes1, which is consistent with NICD. Expression of HIF-1α indicating hypoxia treatment effectively worked. (B) Western blot showed that the expression of Hes1 was down-regulated and the expression of integrin β1 was up-regulated after interference of Notch1 compared with a negative

control (siRNA con). SiRNA interference lasted 6 h, and then cells were cultured for 24 h before harvest. (C) Western blot showed that Jagged-1 (1μg/ml, 24 h) or Dll4 (1μg/ml, 24 h) effectively activate the hypoxia-induced silence of Notch1 signaling.

Figure S4 The regulation of Notch1 signaling and integrin β1 effected HaCaT cells migration. (A) HaCaT cells exposed to indicated treatment were scratch-wounded using Culture-Inserts, whose width of cell-free gap is 500 μm. The results were recorded using a phase-contrast microscope connected to a digital camera from time 0 to 12 h (n = 4 independent experiments). Bar=200 μm. Before 12 h monitoring, each group was treated with indicated treatment for 12 h. As to siRNA groups, 6 h for interference, 12 h cultured under indicated condition. (B)-(D) Wound closure was demonstrated by determining the area covered by keratinocytes immediately after wounding and 12 h later. Each panel represents the wound closure level of each group. The results were calculated by measuring the reduction in the wound bed surface over time using Image J software. *, $P< 0.05$ versus the control group or the group including siRNA con. #, $P< 0.05$ versus the hypoxia group. (E) Western blot analysis showing the expression of integrin β1 after interference with siRNA ITGB1 in HaCaT cells under normoxia (left panel) and hypoxia (right panel) conditions. (F) FAM-siRNA transfected HaCaT cells were observed using fluorescence microscope. Bar=50 μm. (G)-(I) HaCaT cell proliferation was analyzed using Cell Counting Kit-8 (n = 4 independent experiments).

Figure S1

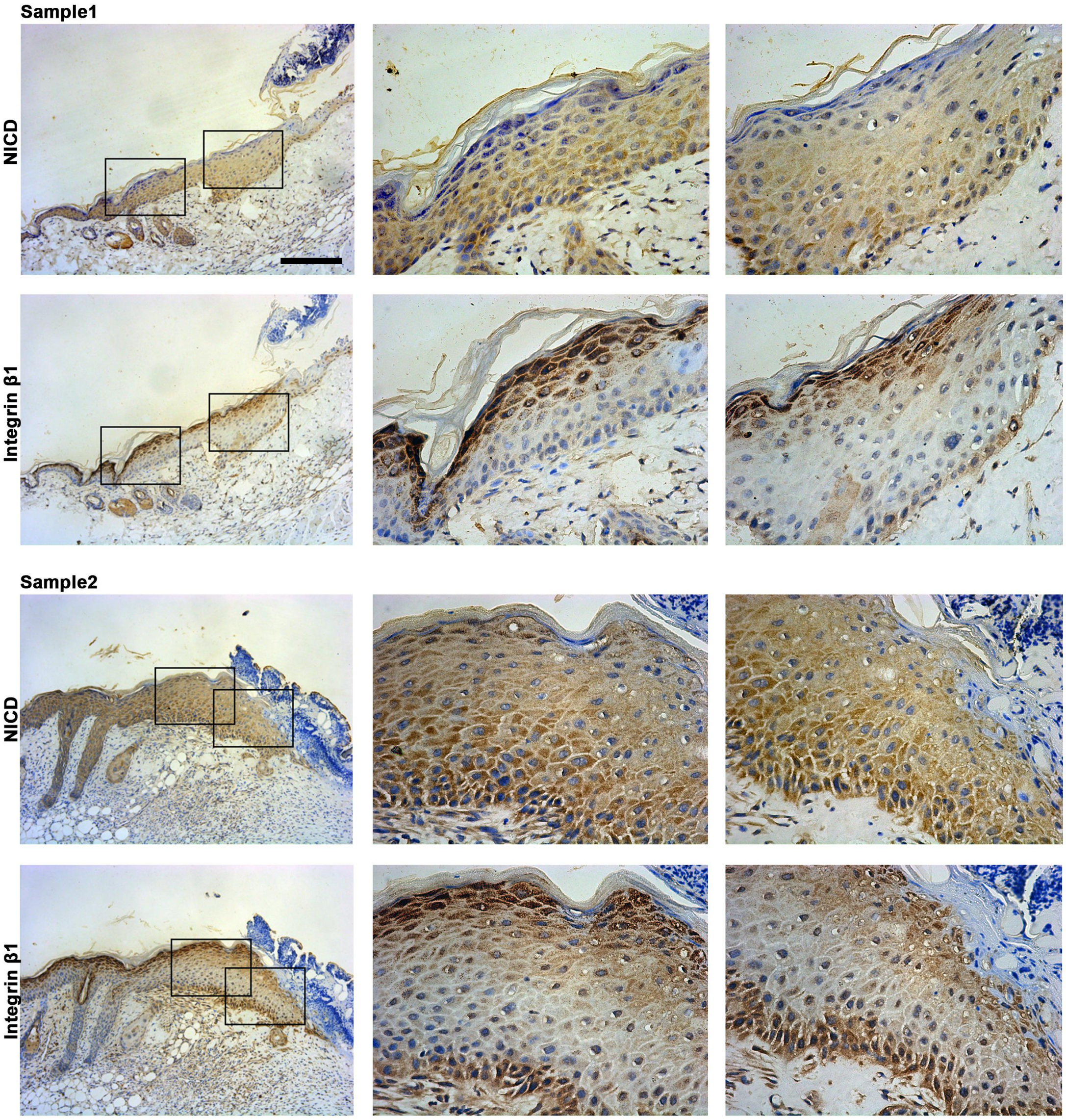

Figure S2

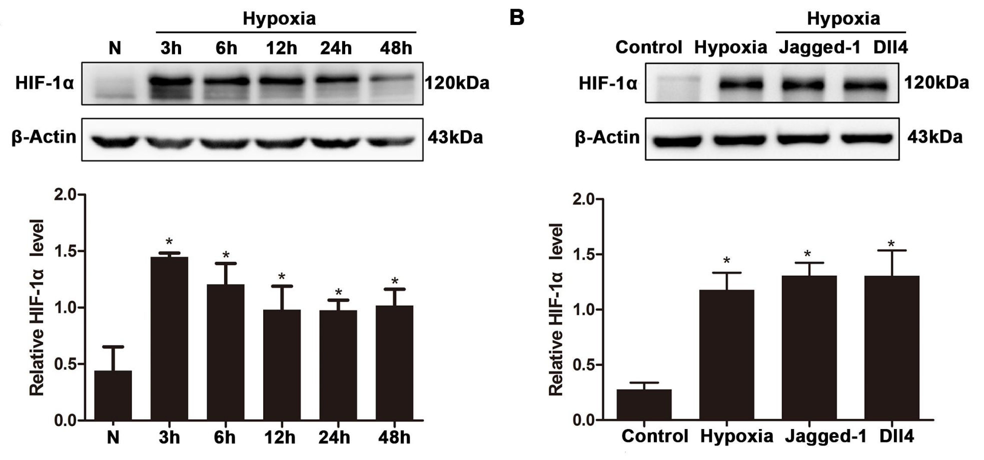

Figure S3

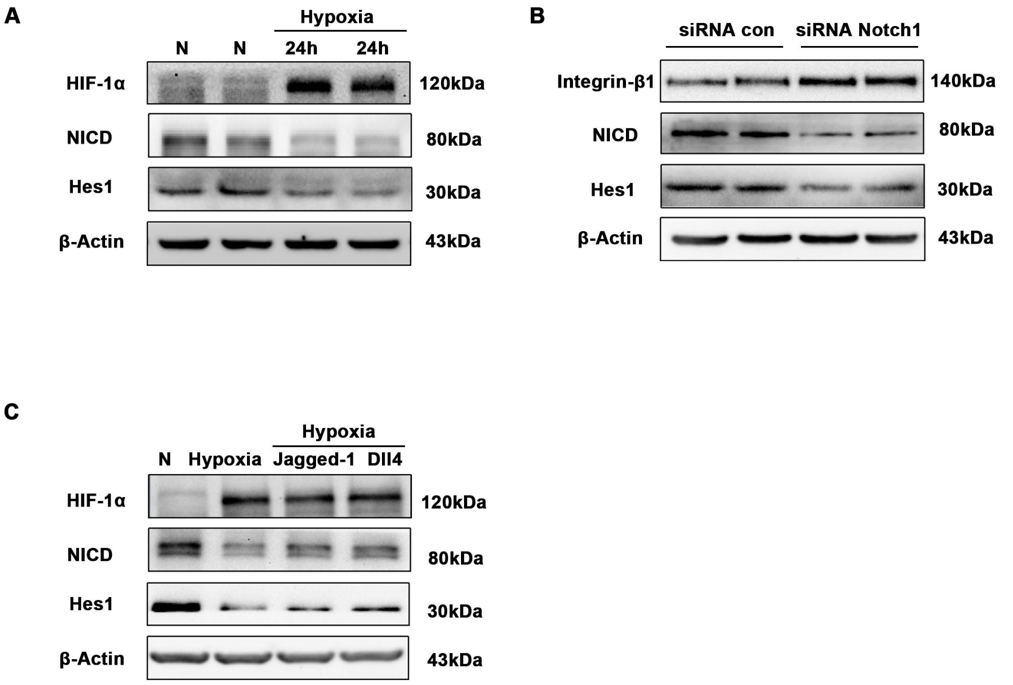

Figure S4

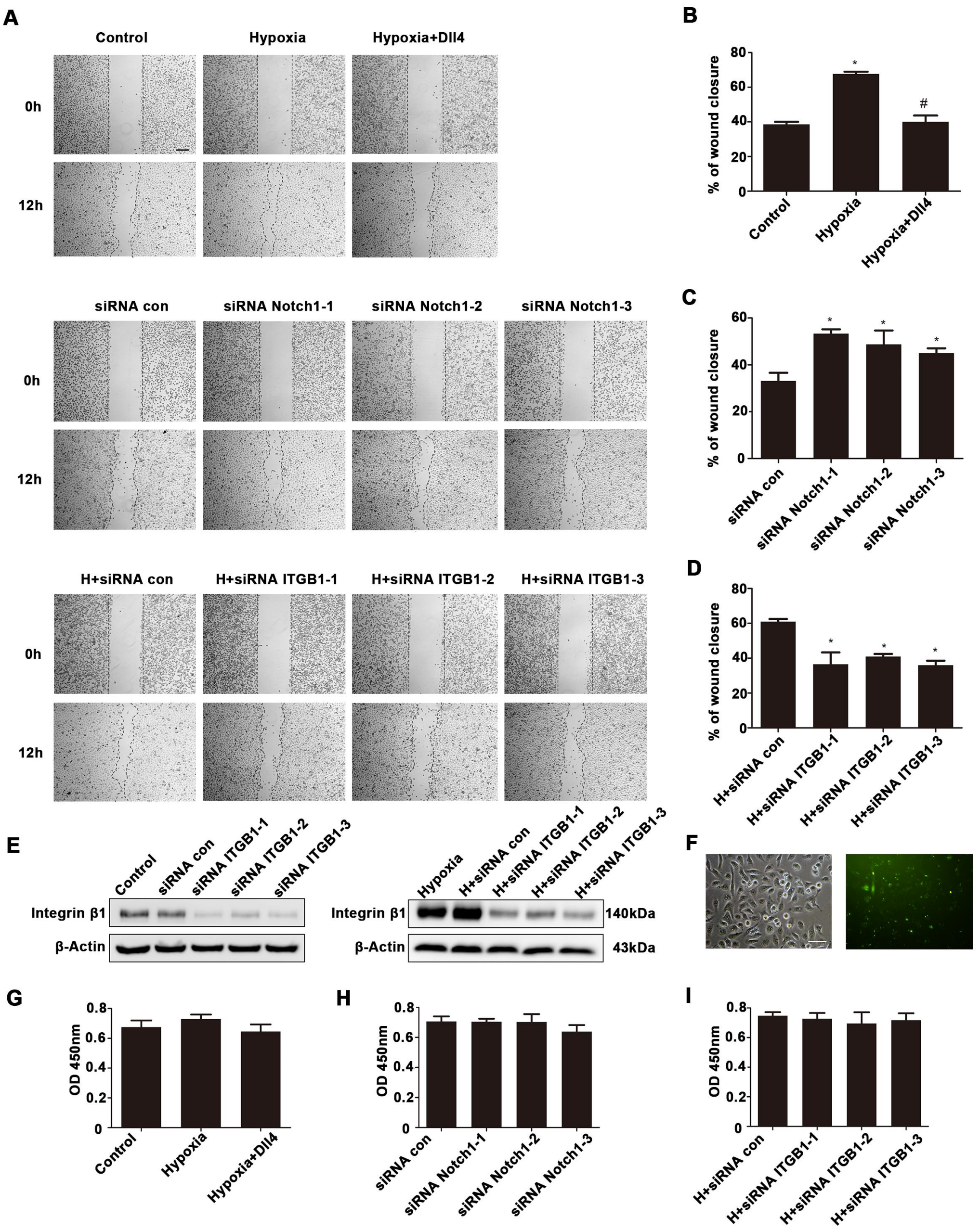
A
Control
Hypoxia
Hypoxia+Dll4
0h
12h
siRNA con
siRNA Notch1-1
siRNA Notch1-2
siRNA Notch1-3
H+siRNA con
H+siRNA ITGB1-1
H+siRNA ITGB1-2
H+siRNA ITGB1-3
B
% of wound closure
C
D
E
Integrin β1
β-Actin
siRNA con
siRNA ITGB1-1
siRNA ITGB1-2
siRNA ITGB1-3
140kDa
43kDa
F
G
OD 450nm
H
I
